# Traditional Tomato Varieties Improve Fruit Quality Without Affecting Fruit Yield Under Moderate Salt Stress

**DOI:** 10.3389/fpls.2020.587754

**Published:** 2020-11-16

**Authors:** Silvia L. R. Meza, Isabel Egea, Isabel L. Massaretto, Belén Morales, Eduardo Purgatto, José M. Egea-Fernández, María C. Bolarin, Francisco B. Flores

**Affiliations:** ^1^Department of Stress Biology and Plant Pathology, CEBAS-CSIC, Espinardo-Murcia, Spain; ^2^Department of Food Science and Experimental Nutrition, Faculty of Pharmaceutical Sciences, Food Research Center, University of São Paulo, São Paulo, Brazil; ^3^Department of Plant Biology, Faculty of Biology, University of Murcia, Murcia, Spain

**Keywords:** secondary metabolites, primary metabolites, sustainable fruit yield, salinity, landraces, *Solanum lycopersicum*, fruit quality improvement

## Abstract

Identification of tomato varieties able to exhibit higher accumulation of primary and secondary metabolites in their fruits is currently a main objective in tomato breeding. One tool to improve fruit quality is to cultivate the plants under salt stress, although improvement of fruit quality is generally accompanied by productivity losses. However, it is very interesting to implement strategies aiming at enhancing fruit quality of tomato by means of growing plants in moderate salt stress that allows for a sustainable fruit yield. The traditional tomato varieties adapted to the Mediterranean environmental constraints may be very attractive plant materials to achieve this goal, given the wide range of fruit quality traits because of their genetic diversity. Here, agronomic responses and fruit quality traits, including primary and secondary metabolites, were analyzed in fruits of two Mediterranean traditional tomato varieties named “Tomate Pimiento” (“TP”) and “Muchamiel Aperado” (“MA”) because of the pepper and pear shape of their fruits, using as reference the commercial cultivar “Moneymaker” (“MM”). Plants were grown without salt (control) and with moderate salt stress (50 mM NaCl), which did not affect fruit yield in any variety. “TP” is of great interest because of its high soluble solids content (SSC) in control, which is even higher in salt, whereas “MA” is very attractive because of its high Brix yield index (SSC × fruit yield), used as overall fruit quality measure. Similitude between both traditional varieties were found for primary metabolism, as they significantly increased sucrose contents compared with “MM” in red ripe fruits from plants in control and, especially, salt stress conditions. The most remarkable difference was the high constitutive levels of total amino acids in “TP” fruits, including the three major free amino acids found in tomato fruit, GABA, glutamate, and glutamine, which even increased under salinity. Regarding secondary metabolites, the most interesting change induced by salinity was the increase in α-tocopherol found in red ripe fruits of both “TP” and “MA.” These results reveal the interest of traditional varieties as sources of genetic variation in breeding because of their improvement of tomato fruit quality without production losses under moderate salt stress.

## Introduction

Salinity affects fruit quality by inducing metabolic changes (Fanciullino et al., 2014; Rouphael et al., 2018). Plants have developed several strategies to counteract increases in environmental salt concentration including accumulation of specific, osmotically active metabolites and specific secondary metabolites, but the effect of salinity can vary depending on the species or variety as well as salt concentration (Albaladejo et al., 2017). Tomato (*Solanum lycopersicum*) is one of the most important horticultural crops worldwide and ranks second after potato in terms of global production and first in terms of yield (FAOSTAT, 2018). The importance of tomato fruit for human health is reflected by its high consumption per capita, and the identification of tomato varieties that accumulate higher levels of primary and secondary metabolites in their fruit is a priority objective (Hou et al., 2020).

A recent review of studies investigating the impact of abiotic stress on primary and secondary metabolism of tomato concluded that understanding tomato responses to salt stress, including the accumulation of metabolites, is critical for maximizing productivity and fruit quality (Quinet et al., 2019). One problem limiting progress in the development of tomatoes containing high levels of health-promoting compounds is genetic erosion (D’Esposito et al., 2017). Modern breeding practices have been shown to alter the tomato fruit metabolome (Zhu et al., 2018). Plant breeding was previously carried out by farmers who selected for specific adaptive traits, which produced the traditional tomato varieties; however, these have been largely replaced by a small number of cultivars in modern plant breeding, which has highly increased the vulnerability of tomato genetic resources (Dwivedi et al., 2017). Because of their closer genetic proximity to modern cultivars than their wild relatives, landraces or traditional varieties that have emerged from adaptive responses to local habitats are a valuable source of many traits of agronomic interest and associated with fruit nutritional quality (Gascuel et al., 2017; Moles et al., 2019).

Plants have a limited supply of resources that are divided between competing physiologic functions, resulting in resource allocation trade-offs (Munns et al., 2020). When plant growth and fruit yield are reduced as a result of exposure to salt stress, these resources may be diverted to metabolite synthesis, resulting in changes in the metabolome that might be different depending on the stress levels. We previously investigated changes in the metabolome induced by high salt stress level (100 mM NaCl) and found that while fruit yield was significantly reduced, fruit quality was improved (Massaretto et al., 2018). From an agronomic standpoint, it is desirable to achieve the latter without sacrificing the former, which may be possible by applying moderate salt stress levels, as controlled application of salt stress in greenhouses has been proposed as an innovative strategy for enhancing crop quality (Toscano et al., 2019). To address this issue, in the present study we carried out comparative metabolomic profiling of tomato plants using two tomato traditional varieties [“Tomate Pimiento” (“TP”) and “Muchamiel Aperado” (“MA”)] that differ in terms of fruit shape and size along with a commercial cultivar [“Moneymaker” (“MM”)] as a reference. The three genotypes maintained fruit yield under moderate salinity (50 mM NaCl); however, under these conditions, the two landraces showed differences in fruit characteristics and metabolite profiles; one of them showed a high amino acid content whereas both increased α-tocopherol content.

## Materials and Methods

### Plant Materials and Growth Conditions

The commercial cultivar “MM” was used as a reference and two traditional tomato varieties, “TP” and “MA,” were selected for their distinct fruit morphology. These traditional varieties were collected in the Southeast area of Spain by the Agroecology Network of the Region of Murcia, Spain (RAERM), and seeds were stored and registered in a seed bank maintained by the Agricultural and Forestry Experimentation Service of the University of Murcia (Egea-Fernandez et al., 2015).

Seeds were germinated in seedbeds in darkness, in a 2:1 *v*/*v* peat/perlite mixture inside a growth chamber under controlled conditions of 28°C and 90% relative humidity (RH). After emergence, seedlings were grown in the same growth chamber under environmental conditions of 18–25°C temperature, 50–70% RH, and a photoperiod of 16 h light/8 h darkness. A photosynthetic photon flux (400–700 nm) of 345 μmol m^2^ s^–1^ was provided at plant level by fluorescent tubes (Luminux Daily Light 58W and Fluora 58W; Osram, Madrid, Spain). During this period, plants were irrigated daily with half-strength Hoagland solution (Hoagland and Arnon, 1950).

A spring–summer culture was maintained in a greenhouse located on the campus of the University of Murcia (Espinardo, Spain) that offered controlled culture conditions. At the four-leaf developmental stage (30 days after sowing), plants were moved to the greenhouse and transplanted; 14 plants per variety were grown in plastic pots containing 18 L of a 2:1 *v*/*v* peat/perlite mixture. The fertigation (i.e., Hoagland) solution was prepared in 2000-L tanks with local irrigation water [electrical conductivity (EC) = 0.9 dS m^–1^], with pH and EC regularly monitored. Plant fertigation was achieved using a drip irrigation system with 3 L h^–1^ drippers. Salt level (50 mM NaCl) was selected from preliminary assays using irrigation waters with increasing NaCl concentrations from 30 to 60 mM, being this salt level where plants were able to maintain fruit production. At the six-leaf stage, when time elapsed from sowing was 45 days, fertigation solution supplemented with 50 mM NaCl was applied to seven plants of each tomato variety for 80 days, while the other seven plants of each variety were irrigated without salt (control condition). Temperature and RH were recorded daily and showed daily fluctuations from 30 to 15°C and 40 to 60% (day/night), respectively. A completely randomized design with seven plants per variety was used for each treatment (0 and 50 mM NaCl).

At the start of salt treatment, plant height, number of leaves, and chlorophyll content and fluorescence were determined to verify the homogeneity of the plants. The latter two physiologic parameters were measured as previously described (Garcia-Abellan et al., 2015).

### Fruit Harvest and Sampling

To determine fruit yield (based on fruit number and fruit weight), ripe fruits from the first to third truss of each plant were collected, weighed, and counted. At the end of the experiment, the shoot of plants was weighed, and the harvest index was estimated as the ratio of fruit yield to total plant biomass (vegetative and reproductive).

To determine fruit quality standard parameters [soluble solids content (SSC) and titratable acidity (TA)], fruits at the red ripe (RR) stage were harvested. Mature-green (MG) fruits that had reached their final size were also harvested for mineral and metabolite analyses. Pericarp of MG and RR fruits was chopped into pieces and immediately frozen in liquid N_2_ and samples stored at −80°C until analyses were completed. Frozen samples were homogenized with a mortar and pestle to proceed with the metabolic analyses. A portion of frozen material was lyophilized for water content analysis, weighting it before and after the freeze-drying process. For each variety, fruit stage, and treatment, three biological replicates of 10 fruits each were analyzed.

### Determination of SSC and Titratable Acidity

For SSC analysis, an aliquot of frozen tomato fruit sample was thawed and filtered through a nylon membrane filter. The supernatant was collected and SSC was measured using a refractometer with automatic temperature compensation (Model PR-101; ATAGO, Tokyo, Japan); the result is expressed as Brix at 20°C. TA was determined in triplicate from the juice by taking 5 g of the thawed fruit pericarp homogenate and adding 45 ml of distilled H_2_O, followed by pH titration with 0.1 M NaOH up to pH 8.1; the result is expressed as grams of citric acid per 100 g of fresh weight (%). Fruit quality indices such as maturation index (SSC/TA ratio) and Brix yield (BY; SSC × fruit yield) were calculated.

### Analysis of Cations by Inductively Coupled Plasma Optical Emission Spectrometry

Fruit samples (pericarp) were lyophilized, milled to powder, and digested for 24 h in a concentrated HNO_3_:HClO_4_ (2:1 *v*/*v*) solution. Na^+^, K^+^, Ca^2+^, and Mg^2+^ contents were determined by inductively coupled plasma optical emission spectrometry (ICP-OES) on an ICAP 6500 DUO/IRIS Intrepid II XLD system (Thermo Fisher Scientific, Waltham, MA, United States). ICP-OES analysis was carried out at the Ionomics Platform of Centro de Edafología y Biología Aplicada del Segura (CEBAS)–Consejo Superior de Investigaciones Científicas (CSIC) (Murcia, Spain).

### Analysis of Primary Metabolites by ^1^H-Nuclear Magnetic Resonance Spectroscopy

Primary metabolites were analyzed as previously described (Choi et al., 2004, 2006), with slight modifications. A 1-ml volume of 1:1 (*v*/*v*) H_2_O:MeOH was added to 50 mg of lyophilized fruit sample (pericarp) and vortexed for 1 min, followed by sonication for 1 min and centrifugation (11,000 × *g* at 4°C for 20 min). The supernatant was collected in a 2-ml microtube and dried in a rotary vacuum evaporator. The dried extract was reconstituted in 800 μl of D_2_O phosphate buffer [100 mM KH_2_PO_4_ (pH 6.0)] containing 0.01% trimethyl silyl propionic acid (TSP) (0.58 mM TSP sodium salt) as an internal standard and vortexed for 1 min. The mixture was centrifuged (16,100 × *g* at 4°C for 5 min) and 600 μl of the supernatant was transferred to nuclear magnetic resonance (NMR) tube for analysis.

All ^1^H NMR spectra were recorded at 298 K on a Bruker AVIII HD 500 NMR spectrometer (500.13 MHz for ^1^H) equipped with a 5-mm CryoProbe Prodigy Broadband Observe cryogenic probe (Biospin; Bruker, Bremen, Germany). The ^1^H spectra were referenced to the TSP signal (δ = 0.00 ppm), whereas ^13^C spectra were referenced to CH-1 resonance of α-D-glucose (δ = 93.10 ppm). For each sample, 32 scans were recorded with the following parameters: 0.126 Hz/point, pulse width = 4.0 μs (30°), and relaxation delay = 1.0 s. Free induction decay was Fourier-transformed with line broadening = 0.5 Hz, Gaussian broadening = 0, and peak-picking sensitivity = 1.0, and peak integral was used for quantitative analysis. Whole-peak intensities in every 0.02 ppm in ^1^H NMR spectra in the range of δ 0.30–12.0 were used as variables. ^1^H NMR spectra were manually corrected for phase and baseline distortions using TOPSPIN v3.2 (Bruker). Peak-fitting on the resultant spectra was performed using an algorithm in Chenomx NMR Suite v8.1 software (Chenomx, Edmonton, AB, Canada) to obtain the concentrations of primary metabolites detected in plant material. The region δ = 4.67–5.15 was discarded to eliminate the effects of imperfect water presaturation. Spectral areas of all buckets were normalized to the weight of extracts used for measurements. The intensities of the selected ^1^H resonances attributable to hydroalcoholic metabolites were measured with respect to the intensity of the TSP signal used as the internal standard at a concentration of 0.58 mM. The metabolite analysis was performed at the Metabolomics Platform of CEBAS–CSIC.

### Analysis of Secondary Metabolites by Ultra-High–Performance Liquid Chromatography

Analysis of carotenoids was carried out as previously described (Massaretto et al., 2018). Briefly, frozen fruit samples (pericarp) (200 mg) were mixed with 100 μl of 30% *w*/*v* NaCl solution and 200 μl of dichloromethane, and vortexed for 1 min. Next, 500 μl of hexane/ether (1:1 *v*/*v*) was added to the mixture, which was stirred for 1 min and centrifuged at 13,000 × *g* at 4°C for 5 min. The supernatant was collected in a 2-ml microtube. This procedure was repeated three times and the organic phases from the repeats were combined. The remaining hexane phase was evaporated under an N_2_ atmosphere. The dried carotenoid extract was reconstituted in 300 μl of the injection solvent composed of acetonitrile (ACN)/MeOH (7:3 *v*/*v*)/acetone (6.7:3.3 *v*/*v*) for LC analysis. All sample solutions were filtered through a Millex 0.2-μm nylon membrane syringe filter (Millipore, Bedford, MA, United States) before injection into an Acquity I Class Ultra Performance LC system connected to a tunable ultraviolet (TUV) detector measuring absorbance at 286 and 450 nm (Waters, Milford, MA, United States). Chromatographic separation was performed on a reversed-phase Acquity UPLC ethylene-bridged hybrid (BEH) C18 column (130 Å, 1.7 μm, 2.1 × 100 mm) (Waters) as previously described (Rivera et al., 2013). Identification was performed by comparing retention times and spectral properties of samples with those of standards (Sigma-Aldrich, St. Louis, MO, United States and CaroteNature, Lupsingen, Switzerland) and reference spectra. Standard stock solutions of major carotenoids present in tomato fruit were prepared using HPLC-grade ethanol (for neoxanthin, violaxanthin, and lutein) or hexane (for phytoene, β-carotene, and lycopene). Before use, aliquots of each stock solution were diluted in the appropriate HPLC-grade solvent and the concentration was determined by UV–visible light absorption at their maximum absorbance wavelengths using the extinction coefficients (ε) described by Rivera and Canela (2012). Calibration was performed with dose–response curves generated using the standard solutions.

The analysis of α-tocopherol was carried out by ultra-high–performance liquid chromatography (UHPLC). The extraction procedure was performed as previously described (Almeida et al., 2011). First, 250 mg of freshly frozen fruit material (pericarp) was extracted with 750 μl of methanol. The resultant mixture was vortexed for 1 min and 500 μl of chloroform was then added, followed by stirring for 1 min and incubation on ice for 10 min in the dark. A 500-μl volume of Tris-buffered saline [50 mM Tris (pH 7.5)/1 M NaCl] was added, and the solution was vortexed for 1 min and centrifuged (3,000 × *g* at 4°C for 5 min). The chloroform phase was recovered and the methanol phase (i.e., the remaining pellet) was re-extracted with 1 ml of chloroform as described previously. The chloroform phases were pooled and adjusted to a final volume of 2 ml and evaporated under an N_2_ atmosphere. The dried α-tocopherol extract was reconstituted in 0.2 ml of injection solvent (2:1 *v*/*v* CH_2_Cl_2_/MeOH) for LC analysis. All sample solutions were filtered through a Millex 0.2-μm nylon membrane syringe filter before injection into the UHPLC instrument.

The α-tocopherol content was determined using a Waters I-Class HPLC system coupled with a TUV detector. Separation was carried out on a normal-phase BEH C18 column (50 mm, 2.1 mm, and 1.7-μm mesh) using an isocratic solvent system (mobile phase) consisting of 3:2 *v*/*v* ACN/MeOH. The column temperature was set at 30°C, the flow rate was 0.5 ml min^–1^, and the total runtime including column equilibration was 5.0 min. Eluting compounds were detected at 292 nm. α-Tocopherol was identified and quantified by comparing the retention time and peak area with those of a standard (Sigma-Aldrich). The stock solution of α-tocopherol was prepared by dissolving 2.54 mg in 1.0 ml HPLC-grade EtOH. Before use, aliquots were diluted in 3:2 *v*/*v* ACN/MeOH and calibration was performed with dose–response curves generated using the standard solutions. The analysis was carried out at the Metabolomics Platform of CEBAS–CSIC.

### Analysis of Chlorophyll Content

Chlorophyll *a* and *b* contents in tomato fruit were determined as previously described (Nagata and Yamashita, 1992) using frozen samples (fruit pericarp). Briefly, 1 g of thawed sample was homogenized with 20 ml of 2:3 *v*/*v* acetone/hexane and centrifuged at 3,000 × *g* for 10 min at 4°C. Absorbance (A) at 663 and 645 nm was measured using a spectrophotometer. Chlorophyll *a* and *b* contents were calculated according to the following equations.

Chlorophylla= 0.999A663-0.0989A645

Chlorophyllb= 1.77A645-0.328A663

### Statistical Analysis

Significant differences between mean values (*p* < 0.05) were evaluated by one-way ANOVA, Student’s *t*-test, or Tukey’s test. Statistical analyses were performed using Minitab v19.0 software (Minitab, State College, PA, United States). A principal component analysis (PCA) biplot and heatmap were generated from data matrices and used to ascertain the overall variability across cultivars and treatments for each fruit stage (MG and RR). Multivariate analysis was performed using the Metaboanalyst 4.0 server (Chong et al., 2019). Raw data were first normalized according to median values, log2-transformed, mean-centered, and divided by the root-mean-square deviation of each variable (Pareto scaling). A univariate analysis of fold change was also performed using Metaboanalyst 4.0 server to evaluate significant differences between accumulated metabolites in “TP” and “MA” versus “MM” tomato fruits.

## Results

### Agronomic Response and Fruit Organoleptic Attributes of Traditional Versus Commercial Tomato Varieties

**FIGURE 1:**
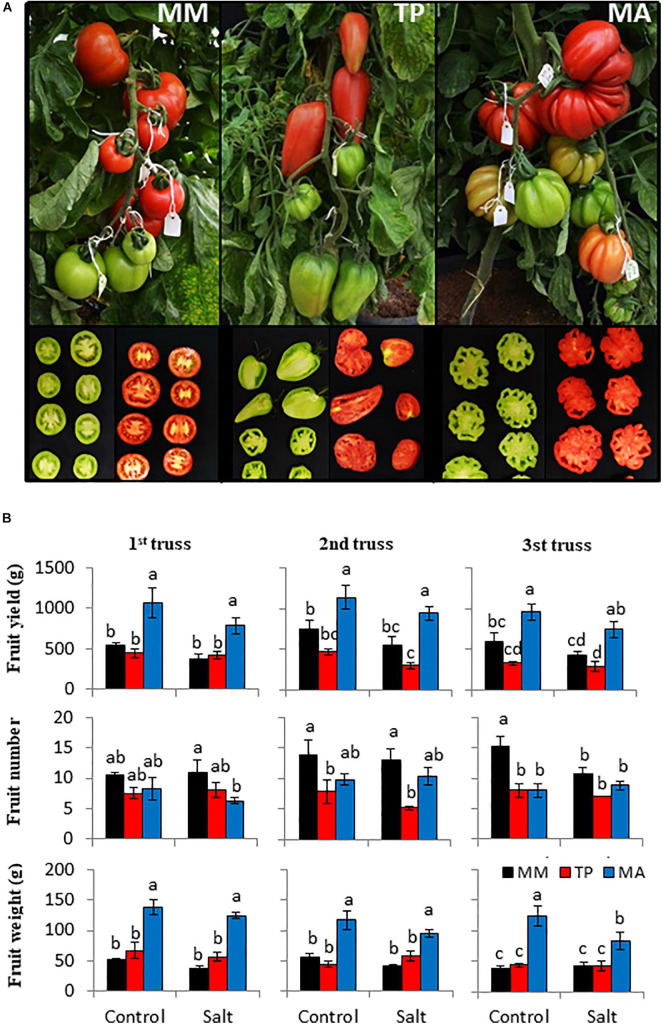
Agronomic response of “Moneymaker” (MM) and two traditional varieties, “Tomate Pimiento” (TP) and “Muchamiel Aperado” (MA), to moderate salinity levels (50 mM NaCl). **(A)** Representative images of the fruits from the first truss showing the different fruit morphological characteristics of both landraces with respect to MM. **(B)** Fruit yield (g FW), fruit number, and fruit weight (g FW) from the first three trusses of plants grown in control and salt for 60, 70, and 80 days of 50 mM NaCl treatment. Values are expressed as means ± SE of seven plants per variety. Different letters indicate statistically significant differences according to Tukey’s test (*p* < 0.05).

The traditional tomato varieties “TP” and “MA” were selected for the distinct morphology of their fruits; “TP” fruits are mid-sized and elongated, and similar in shape to bell peppers, whereas “MA” ones have large ribbed fruits that are pear-shaped with numerous locules. In contrast, the commercial cultivar “MM” used as reference has round-shaped fruits with two locules (Figure 1A). Vegetative development was similar in “TP,” “MA,” and “MM” before the application of salt stress (Supplementary Figure 1A); the only difference was the greater plant height of “MA” owing to a larger internode distance, although leaf number was similar for the three genotypes (Supplementary Figure 1B). Moreover, leaf chlorophyll content and chlorophyll fluorescence, two important physiologic traits related to salinity tolerance, were similar for “TP,” “MA,” and “MM” (Supplementary Figure 1B). Given these characteristics, both landraces represent excellent materials to study the response of salt stress at the reproductive level.

**FIGURE 2:**
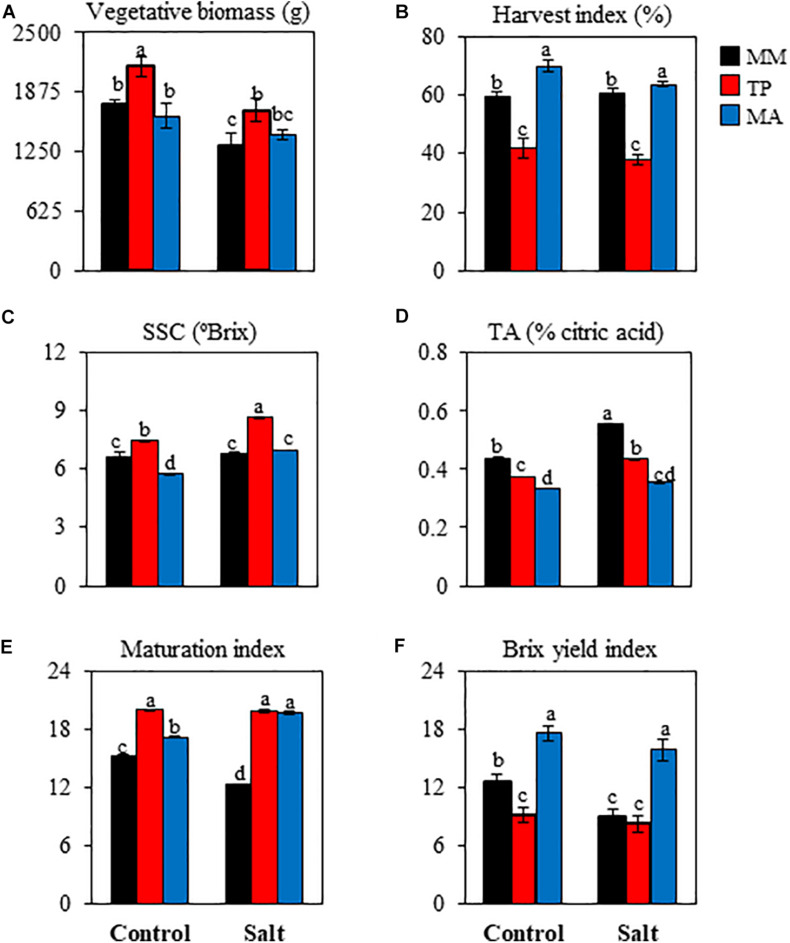
Agronomic and organoleptic quality parameters in red ripe fruits of tomato “Moneymaker” (MM) and two traditional varieties, “Tomate Pimiento” (TP) and “Muchamiel Aperado” (MA), from plants grown in control and salt (50 mM NaCl). **(A)** Vegetative biomass, **(B)** harvest index, **(C)** soluble solid content (SSC), **(D)** titratable acidity (TA), **(E)** Maturation Index (SSC/TA), **(F)** Brix yield index (SSC × fruit yield). For agronomic data, seven plants were individually harvested; for quality traits, three biological replicates of 10 fruits each were used. Values are expressed as means ± SE. Different letters indicate statistically significant differences according to Tukey’s test (*p* < 0.05).

Given that salt stress can variably affect the fruit yield of each truss and because the duration of exposure to salt treatment is longer for the upper trusses, fruit yield and its components (fruit number and fruit weight) were estimated for the first three trusses (Figure 1B). Although there was a clear trend of decreasing fruit yield with exposure to moderate salinity (50 mM NaCl), especially in “MM” and “MA,” there were no significant differences in yield between the control and salt conditions in any genotype. “MA” had the highest fruit yield because it had the highest fruit weight, which was only significantly affected by salinity at the third truss. Regarding fruit number, neither landrace was affected by salinity at any truss, and in “MM” it was only significantly reduced at the third truss. These results show that the moderate salt stress applied in our study had a slight effect on the reproductive development of tomato plants without significantly altering fruit yield and harvest index (Figures 1B, 2B). However, harvest index varied across genotypes: “TP” had the lowest value, which was attributable to its highest vegetative biomass (Figure 2A), whereas the high harvest index of “MA” was mainly due to its highest fruit yield (Figure 1B).

Regarding fruit quality parameters, SSC was significantly higher in RR fruits of “TP” but significantly lower in RR fruits of “MA” compared with “MM” under control treatment (Figure 2C). Salt stress increased SSC in RR fruits of both traditional varieties relative to the control treatment, whereas in “MM,” SSC values were similar under both conditions. TA was lower in RR fruits of both traditional varieties compared with the value in “MM,” with the difference being even greater under salt stress (Figure 2D). Interestingly, the maturation index (MI; i.e., SSC/TA ratio) was higher in both traditional varieties than in “MM” under control as well as salt treatment (Figure 2E). To better quantify fruit quality, we calculated BY, a composite index representing the weight of SSC per plant that has been used as a measure of commercial quality. BY was strikingly high in “MA” (Figure 2F), with similar values in fruits obtained from control and salt-stressed plants. Thus, both traditional tomato varieties have similar fruit characteristics including a high MI, but differ in SSC and BY (elevated in “TP” and “MA,” respectively).

### Physiologic Changes Induced by Salt Stress in Tomato Fruit

Given that physiologic and metabolic changes can vary during ripening, fruits were analyzed at two developmental stages, namely MG and RR. Notably, water content was lower under salt stress in all genotypes at both fruit stages (Supplementary Table 1). “MA” showed the smallest reduction in water content with moderate salinity in RR fruits; at the MG stage, “TP” had the lowest water content under the control treatment and its reduction under salt stress was also smaller than for “MA” and “MM.”

As increases in solute levels reflect both active solute accumulation and the concentration effect due to dehydration when values are determined based on fresh weight or water basis, we expressed cations and metabolites contents based on dry weight to avoid the effect of solutes increase exclusively due to a concentration effect. K^+^ contents were similar between the two landraces and “MM” at both stages of ripening and did not change with salt stress (Supplementary Table 1). As expected, Na^+^ significantly increased with salinity in the three genotypes, but the accumulation was significantly higher in MG and RR fruits of “MA” than in the fruits of “TP” and “MM”, suggesting that this traditional variety accumulates more solutes to avoid a reduction in water content. Ca^2+^ and Mg^2+^ contents were similar in the three genotypes and were unaffected by salinity. The most important differences were observed for the Ca^2+^/Mg^2+^ ratio in RR fruits, which was significantly higher in “MM” than in the two traditional varieties, especially under salt stress (Supplementary Table 1).

### Primary Metabolites in MG and RR Fruits

**FIGURE 3:**
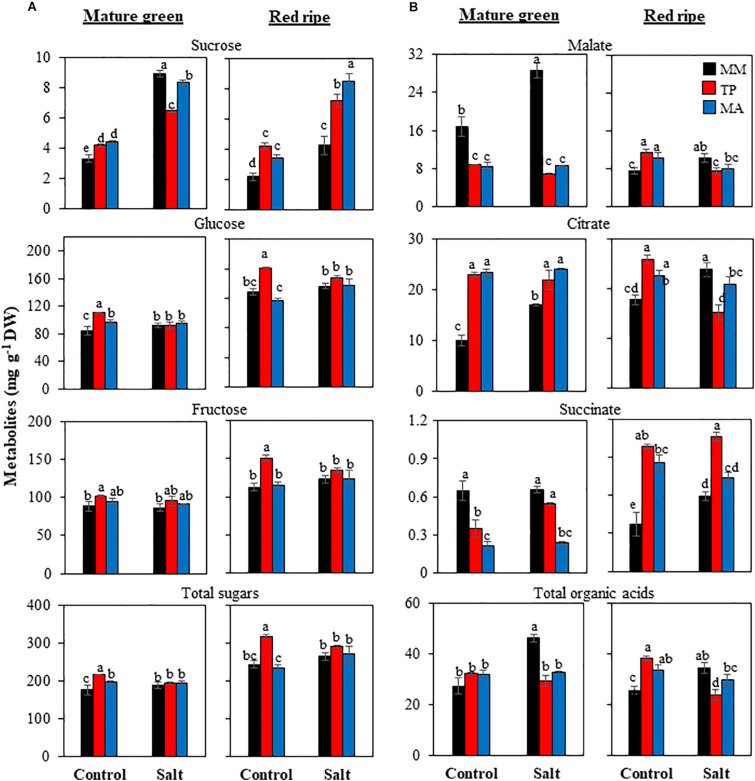
Accumulation of sugars **(A)** and organic acids **(B)** in mature green and red ripe fruits of “Moneymaker” (MM) and two traditional varieties, “Tomate Pimiento” (TP) and “Muchamiel Aperado” (MA), from plants grown in control and salt (50 mM NaCl). Three biological replicates of 10 fruits each were used. Values are expressed as means ± SE. Different letters indicate statistically significant differences according to Tukey’s test (*p* < 0.05).

**FIGURE 4:**
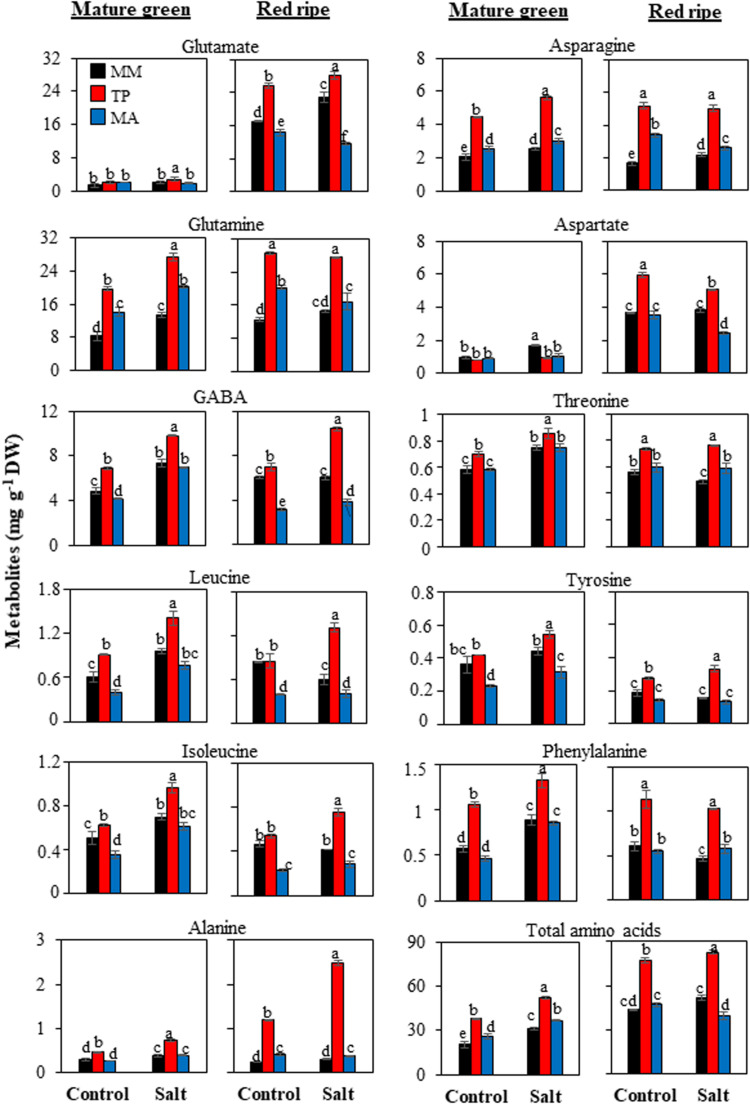
Accumulation of amino acids in mature green and red ripe fruits of “Moneymaker” (MM) and two traditional varieties, “Tomate Pimiento” (TP) and “Muchamiel Aperado” (MA), from plants grown in control and salt (50 mM NaCl). Three biological replicates of 10 fruits each were used. Values are expressed as means ± SE. Different letters indicate statistically significant differences according to Tukey’s test (*p* < 0.05).

The levels of sugars and organic acids (Figure 3) as well as amino acids (Figure 4) were analyzed in MG and RR fruits. Sucrose significantly increased with salinity in MG fruits in the three genotypes, with “MM” showing the greatest increase. The same trend was observed in RR fruits, but at this fruit stage the increases were much higher in both traditional varieties than in “MM” (up to 150% for “MA” fruits) (Figure 3A). The most obvious changes regarding hexoses (glucose and fructose) were the high levels in RR fruits of “TP” when plants were grown in under the control treatment, with the differences between genotypes disappearing under salt stress. The total sugar content reflected changes in the most abundant sugars (hexoses) (Figure 3A). The organic acids malate and citrate showed opposite trends between the two landraces and the commercial cultivar at the MG stage: malate levels were much lower in both traditional varieties than “MM,” especially under salt treatment, whereas citrate levels were significantly higher. Succinate showed a similar trend to malate (Figure 3B). Total organic acid contents in fruits at the MG stage were comparable across the three genotypes under the control condition, likely because any differences in the levels of individual organic acids were abolished through compensatory mechanisms; this did not occur under salt stress, as organic acid level was significantly lower in both traditional varieties compared with “MM” due to the extremely high malate content of MG fruits in the latter. Organic acid levels were higher in RR fruits of both traditional varieties compared with those of “MM” from control plants and the opposite was true in RR fruits from salt-treated plants, with the exception of succinate levels (Figure 3B).

The most remarkable difference across genotypes in terms of primary metabolites was the amino acid profile: “TP” accumulated more amino acids in its fruits than either “MA” or “MM” (Figure 4). “TP” fruits showed the highest accumulation not only of major free amino acids such as glutamate, glutamine, and γ-aminobutyric acid (GABA) but also of minor ones including aromatic amino acids involved in the shikimate pathway such as tyrosine and phenylalanine. In “MA” fruits, total amino acid content was lower than in “TP” fruits but higher than in “MM” fruits at the MG stage, whereas at the RR stage the levels were similar (control treatment) or even lower (salt treatment) than in “MM” fruits (Figure 4).

**FIGURE 5:**
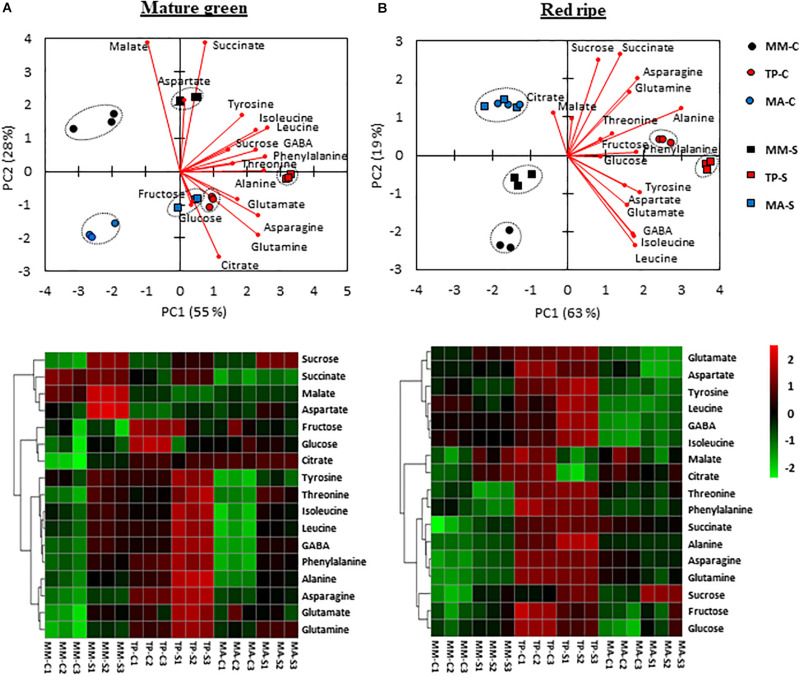
Changes in the profile of primary metabolites (sugars, organic acids, and amino acids) in mature green and red ripe fruits of “Moneymaker” (MM) and two traditional varieties, “Tomate Pimiento” (TP) and “Muchamiel Aperado” (MA), from plants grown in control (C) and salt (50 mM NaCl) (S). Non-supervised principal component analysis (PCA biplot) (top graphics) and heatmap analysis (bottom graphics) representing the major sources of variability in mature green **(A)** and red ripe fruits **(B)**. Color scale represents the variation in the relative concentration of compounds, from high (red) to low (green) contents.

To determine whether separation in primary metabolism between genotypes and at what developmental stage this occurred, we carried out a PCA on all primary metabolites in MG and RR fruits (Figure 5). In the former, the PCA biplot showed clear separation of both traditional varieties from “MM,” with PC1 and PC2 accounting for 55 and 28% of the total variance, respectively (Figure 5A). Amino acids significantly contributed to the separation of samples by PC1 whereas organic acids were the most important metabolites for separation by PC2, with malate and succinate having higher coefficients in “MM,” and citrate having a higher coefficient in the two landraces. Salt stress had a marked effect on the metabolite profiles of the three genotypes: the metabolite contents increased but maintained their distinct metabolic signatures, as revealed by heatmap analysis (Figure 5A). PC1 accounted for a higher percentage of the total variation (63%) at the RR stage than at the MG stage and clearly separated the “TP,” which had higher positive coefficients for amino acids than the other genotypes (Figure 5B). Furthermore, RR fruits from control and salt-treated plants were closer in “MM” and “TP” and in the case of “MA” no separation was observed, suggesting that the metabolic profiles of RR stage is influenced to a greater extent by genotype than by salt stress (Figure 5B). The PCA-biplot and heatmap analyses clearly indicated that sucrose was the main trait responsible for the separation of both traditional tomato varieties from “MM.”

### Secondary Metabolites in MG and RR Fruits

**FIGURE 6:**
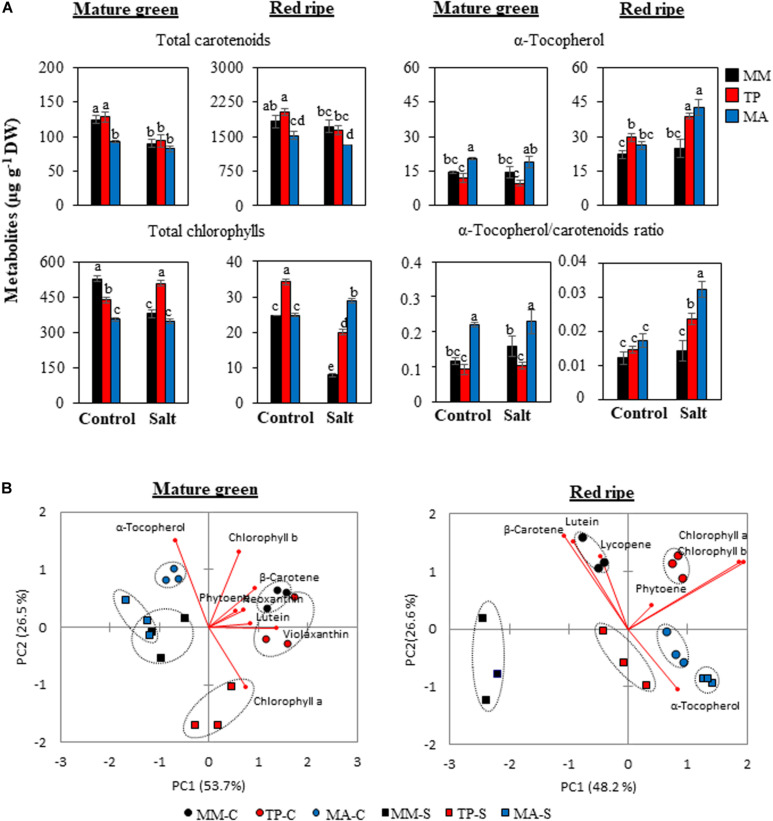
Secondary metabolites in mature green and red ripe fruits of “Moneymaker” (MM) and two traditional varieties, “Tomate Pimiento” (TP) and “Muchamiel Aperado” (MA), from plants grown in control (C) and salt (50 mM NaCl) (S). **(A)** Accumulation of total carotenoids, total chlorophylls, and α-tocopherol, and α-tocopherol/carotenoid ratio. Three biological replicates of 10 fruits each were used. Values are expressed as means ± SE. Different letters indicate statistically significant differences according to Tukey’s test (*p* < 0.05). **(B)** Graphical representation of principal component analysis representing the major sources of variability for secondary metabolites in mature green and red ripe fruits.

As carotenoids, tocopherols, and chlorophylls share a common precursor [geranyl geranyl diphosphate (GGPP)], there is an inverse relationship among these metabolites, which is evident in the PCA biplots of MG and RR fruits (Figure 6B), although the patterns differed according to developmental stage. The α-tocopherol content was high in MG fruits of “MA” whereas the opposite trend was observed for carotenoids and chlorophylls, whereas in “TP” only chlorophyll content was elevated, especially chlorophyll *a* level in MG fruits from salt-treated plants (Figure 6A and Supplementary Table 2). RR fruits of both landraces showed a greater than 50% increase in α-tocopherol content compared with “MM” under moderate salinity, which was reflected by their high α-tocopherol/carotenoid ratios (Figure 6A). Total carotenoid levels were similar in “MM” and “TP” fruits but were comparatively lower in “MA” fruits except at the MG stage in salt-treated plants. This was mainly attributable to the high β-carotene and lycopene levels in MG and RR fruits, respectively (Figure 6A and Supplementary Table 2).

**FIGURE 7:**
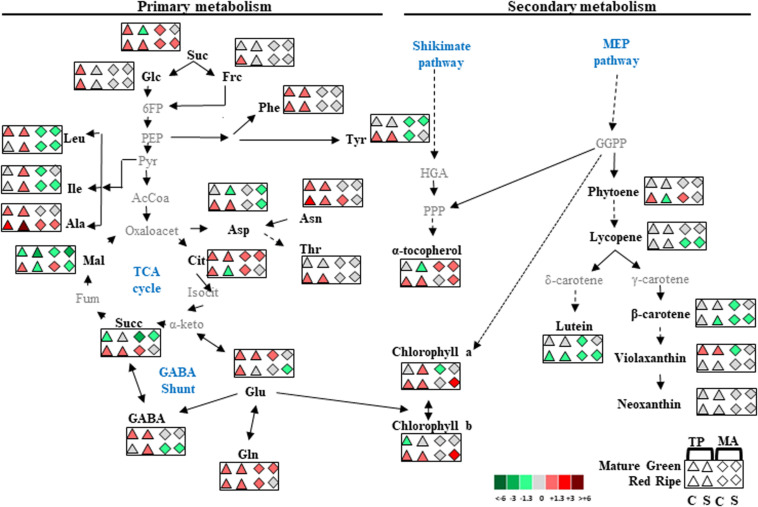
Global overview of metabolic changes occurring in mature green and red ripe fruits of “Tomate Pimiento” (TP) and “Muchamiel Aperado” (MA), from plants grown in control (C) and salt (50 mM NaCl) (S). Data were normalized to “Moneymaker” (MM). Only those metabolites showing upregulation or downregulation in each traditional variety higher 1.3-fold than MM are shown. Color scale is used to display the different amount of metabolite in terms of fold change relative to the level in the appropriate control. Suc, sucrose; Glc, glucose; Fru, fructose; 6FP, fructose-6-phosphate; PEP, phosphoenolpyruvate; Phe, phenylalanine; Tyr, tyrosine; Leu, leucine; Ile, isoleucine; Ala, alanine; Asp, aspartate; Asn, asparagine; Thr, threonine; Pyr, pyruvate; HGA, homogentisic acid; GGPP, geranylgeranyl diphosphate; PPP, phytyl diphosphate; AcCoA, acetyl-CoA; Oxaloacet, oxaloacetate; Cit, citrate; Isocit, isocitrate; α-keto, α-ketoglutarate; Succ, succinate; Fum, fumarate; Mal, malate; Glu, glutamate; Gln, glutamine; GABA, gamma-aminobutyric acid.

There was a very small separation between control and salt treatments for “MA” fruits at the RR stage whereas for “MM” and “TP,” salt stress had a significant effect on the metabolite composition of RR fruits, similar to the changes observed in MG fruits (Figure 6B). This set of results suggests improvement of the metabolic profile occurs in both traditional varieties, but the processes operating in each one seem to be different. Based on our observations, we present a model of global changes in primary and secondary metabolic profiles in MG and RR fruits of traditional tomato varieties compared with the commercial cultivar coming from control and salt treatments (Figure 7).

## Discussion

### Moderate Salt Stress Improves Organoleptic Fruit Quality of Tomato Without Affecting Fruit Yield

The application of controlled abiotic stress can improve the sensory and nutritional value of fruits (Toscano et al., 2019). As changes in tomato fruit quality in response to abiotic stress are cultivar dependent (Quinet et al., 2019), we compared two traditional varieties, “TP” and “MA,” differing widely in terms of fruit shape and size between them and compared with the commercial cultivar “MM” (Figure 1A) to determine whether fruit yield and/or quality are affected when plants were grown at moderate salt stress (50 mM NaCl). In tomato varieties with indeterminate growth, fruits at different positions on different trusses are always at different developmental stages (Ripoll et al., 2016); therefore, the effects of salt stress can vary for each truss. However, we observed similar fruit yields for the first and third trusses of all three tomato genotypes at moderate salinity, with a trend for slightly higher yield for the second truss (Figure 1B). It is worth noting that tomato varieties with small-to-medium fruit size exhibit greater improvements in fruit quality while maintaining the same fruit yield under abiotic stress (Albert et al., 2016), but this link is very rare to observe in varieties with a large fruit size such as “MA.” This provides an opportunity to investigate the possibility of increasing the metabolites content of tomato fruits through irrigation with saline water without negatively affecting fruit yield.

Interestingly, both traditional varieties showed improved organoleptic fruit quality compared with “MM,” although the quality characteristics differed. “TP” is a variety of great interest because of its high SSC (Figure 2C), which is considered an important determinant of tomato fruit organoleptic quality (Hou et al., 2020). Meanwhile, “MA” is interesting because of its high BY index (Figure 2F), which is a measure of overall fruit quality (Rowland et al., 2020). One feature that improved in both landraces was MI, although this index was independent of salinity in “TP” and increased with salinity in “MA,” unlike in “MM” (Figure 2E). In general, increases in SSC associated with salt stress reflect concentration effects resulting from a decreased amount of water in the fruit (Albert et al., 2016), but this was not the case in our study as the reductions in fruit water content induced by salinity were minimal and very similar between “MM” and “TP” (∼2%), and even lower in “MA” (Supplementary Table 1). This along with the fact that salinity reduced vegetative biomass by >20% in “MM” and “TP” (Figure 2A) suggests that the tomato plants buffered the osmotic effect of salinity by limiting their vegetative growth and reallocating water and solutes to the fruits (Osorio et al., 2014). Regarding inorganic solutes, the most significant change was in the Ca^2+^/Mg^2+^ratio, which is often correlated with organoleptic quality traits such as SSC and TA (Gerendás and Führs, 2013). In fact, our results suggest an inverse relationship between the Ca^2+^/Mg^2+^ ratio and MI (SSC/TA ratio) in RR fruits (Figure 2E and Supplementary Table 1) because the decreases in the former (by ∼40%) were accompanied by parallel increases in the latter in both traditional varieties compared with “MM.”

### TP Fruits Exhibit a Remarkable Accumulation of Amino Acids

Primary metabolism is essential for plant growth but is also a major contributor to fruit quality; thus, further advances in its understanding are needed to identify future strategies for manipulation of fruit metabolism (Beauvoit et al., 2018). The first major metabolic change in tomato fruit was sucrose content at the RR stage, as both traditional varieties significantly showed increased sucrose levels under control condition and especially under salt treatment (Figure 3A). A similar response has been reported in two different tomato landraces, in which fruit yield was reduced when plants were irrigated with 100 mM NaCl (Massaretto et al., 2018); this implies that fruit quality is related to a greater ability to accumulate sucrose. “MM” fruits had a very high level of malate at the MG stage compared with “TP” and “MA” whereas the opposite was observed for citrate (Figure 3B), reflecting the “non-cyclic” partial tricarboxylic acid cycle in which one branch produces citrate while the other produces malate (Igamberdiev and Eprintsev, 2016). It was reported that changes in malate metabolism affect fruit quality, as tomato varieties with a high malate content had a low SSC at harvest (Centeno et al., 2011).

There was a clear separation between genotypes in the PCA of MG and especially RR fruits, with a greater abundance of metabolites in “TP” than in “MM” and “MA” (Figure 5). The most remarkable difference was in total amino acid content, which was highest in “TP” and increased with salt treatment (Figure 4). This included the free amino acids involved in the GABA shunt, which plays a major role in primary carbon and nitrogen metabolism and is especially important in certain physiologic situations such as plant stress and fruit ripening (Aghdam and Fard, 2017). The GABA shunt is involved in salt stress tolerance in tomato plants (Bao et al., 2014). Interestingly, we observed that GABA content showed the highest increase with salt stress in MG and RR fruits of “TP” (Figure 4). GABA is a four carbon non-protein amino acid that has received much attention as a health-promoting functional compound (Takayama and Ezura, 2015; Zhao et al., 2018). Glutamate and glutamine—the other two amino acids involved in the GABA shunt—were the two major amino acids detected at the RR stage in every genotype, with especially high levels in “TP” (Figure 4). Notably, glutamine was the only amino acid that showed similar profiles in both landraces in MG and RR stages and culture conditions, with levels higher than “MM” (Figure 7).

### α-Tocopherol Content in Tomato Fruit Is Increased Under Salt Stress in the Two Traditional Varieties

Carotenoids, tocopherols, and chlorophylls share a common precursor (GGPP) produced by the methylerythritol 4-phosphate pathway. Because of this metabolic cross-talk, changes in one of these compounds can affect the biosynthesis of the others (Quadrana et al., 2013; Almeida et al., 2015). We observed a marked increase in α-tocopherol content (>50%) in RR fruits of both traditional varieties under salt treatment compared with fruits from “MM” plants under the same conditions (Figure 6A). However, the metabolic processes operating in the two landraces appear to differ, as the increase in α-tocopherol in “MA” was accompanied by a decrease in carotenoids, whereas “TP” showed similar carotenoid levels as “MM” despite an increase in α-tocopherol (Figure 6 and Supplementary Table 2). Given that α-tocopherol is synthesized from phytyl diphosphate (PPP) generated by GGPP and homogentisate (HGA) from the shikimate pathway (Almeida et al., 2015), we speculate that the accumulation of α-tocopherol in RR fruits of “TP” is related to that of amino acids such as tyrosine and phenylalanine from the shikimate pathway, which does not occur in “MA” and “MM” fruits (Figures 4, 7).

The mechanism of salt tolerance in plants subjected to salinity depends on the level of salt stress (Muñoz-Mayor et al., 2012), which along with genetic background determines the pathways that are induced and specific metabolites that accumulate as a result. In our previous study of two other tomato varieties subjected to a higher salt stress level (100 mM NaCl), the main secondary metabolites that were increased were carotenoids (Massaretto et al., 2018), whereas in the present work, α-tocopherol showed the greatest change in tomato plants under a lower intensity of salt stress (50 mM NaCl). An unsolved question is whether this is only a result of differences in genotype or different salt stress levels may play a role. In any case, it is interesting to highlight the benefits of increased levels of α-tocopherol in fruits of both varieties because this compound is a potent antioxidant and suboptimal amounts in the diet have been related to cardiovascular disease, some types of cancer, and impaired immune function (Saini et al., 2017).

## Conclusion

In conclusion, the two traditional varieties used in this study are attractive sources of genetic variation in breeding because of their improvement of tomato fruit quality under moderate salt stress. These traditional varieties were collected in the semiarid area of Spanish Southeast, featured by its very hot summers and the use of saline waters for irrigation due to the scarcity of hydric sources. Therefore, they constitute a rich reservoir of valuable tomato traditional varieties for breeding focused in improvement of fruit quality of tomato growing in a scenario of climate change. It is interesting to remark that development of new cultivars on the basis of their contents in health-promoting compounds is still very limited. Finally, the fact that α-tocopherol level in fruit nearly doubled in “TP” and “MA” plants relative to “MM” upon irrigation with 50 mM NaCl while fruit yield was unaffected demonstrates that cultivating these landraces under moderate salt stress is an effective agronomic strategy for improving the nutritional value of tomato fruit.

## Data Availability Statement

The original contributions presented in the study are included in the article/Supplementary Materials, further inquiries can be directed to the corresponding author/s.

## Author Contributions

SLRM, IE, and BM performed the experiments. SLRM, IE, and ILM collaborated in the design of the experiments and data analyses. EP, JME-F, MCB, and FBF conceived the project and research, and supervised the experiments. MCB and FBF wrote the manuscript. All authors contributed to the article and approved the submitted version.

## Conflict of Interest

The authors declare that the research was conducted in the absence of any commercial or financial relationships that could be construed as a potential conflict of interest.

## Supplementary Material

Supplementary Figure 1 Phenotypes of “Moneymaker” (MM) and both traditional plant varieties, “Tomate Pimiento” (TP) and “Muchamiel Aperado” (MA), just before salt treatment (50 mM NaCl), after 45 days from sowing. **(A)** Representative images of plants and **(B)** traits of plant development (plant height and leaf number) and leaf chlorophyll content and fluorescence just before starting the salt treatment (50 mM NaCl). Values are expressed as means ± SE of 14 plants per genotype. Different letters indicate statistically significant differences according to Tukey’s test (*p* < 0.05).

Supplementary Table 1 Water percentage and cation contents (mg g^–1^ DW) in mature green and red ripe fruits of “Moneymaker” and two traditional varieties, “Tomate Pimiento” and “Muchamiel Aperado,” from plants grown in control and salt (50 mM NaCl). Three biological replicates of 10 fruits each were used. Values are expressed as means ± SE. Different letters indicate statistically significant differences according to Tukey’s test (*p* < 0.05).

Supplementary Table 2 Carotenoids and chlorophylls contents (μg g^–1^ DW) in mature green and red ripe fruits of “Moneymaker” and two traditional varieties, “Tomate Pimiento” and “Muchamiel Aperado,” from plants grown in control and salt (50 mM NaCl). Three biological replicates of 10 fruits each were used. Values are expressed as means ± SE. Different letters indicate statistically significant differences according to Tukey’s test (*p* < 0.05).
