# Supplementary material for: Traditional Tomato Varieties Improve Fruit Quality Without Affecting Fruit Yield Under Moderate Salt Stress
Source: Front Plant Sci. 2020 Nov 16;11:587754. doi: 10.3389/fpls.2020.587754 (PMC7701295; doi:10.3389/fpls.2020.587754)
Supplement: Supplementary Figure 1 — Phenotypes of “Moneymaker” (MM) and both traditional plant varieties, “Tomate Pimiento” (TP) and “Muchamiel Aperado” (MA), just before salt treatment (50 mM NaCl), after 45 days from sowing. (A) Representative images of plants and (B) traits of plant development (plant height and leaf number) and leaf chlorophyll content and fluorescence just before starting the salt treatment (50 mM NaCl). Values are expressed as means ± SE of 14 plants per genotype. Different letters indicate statistically significant differences according to Tukey’s test (p < 0.05). [file Data_Sheet_1.PDF]

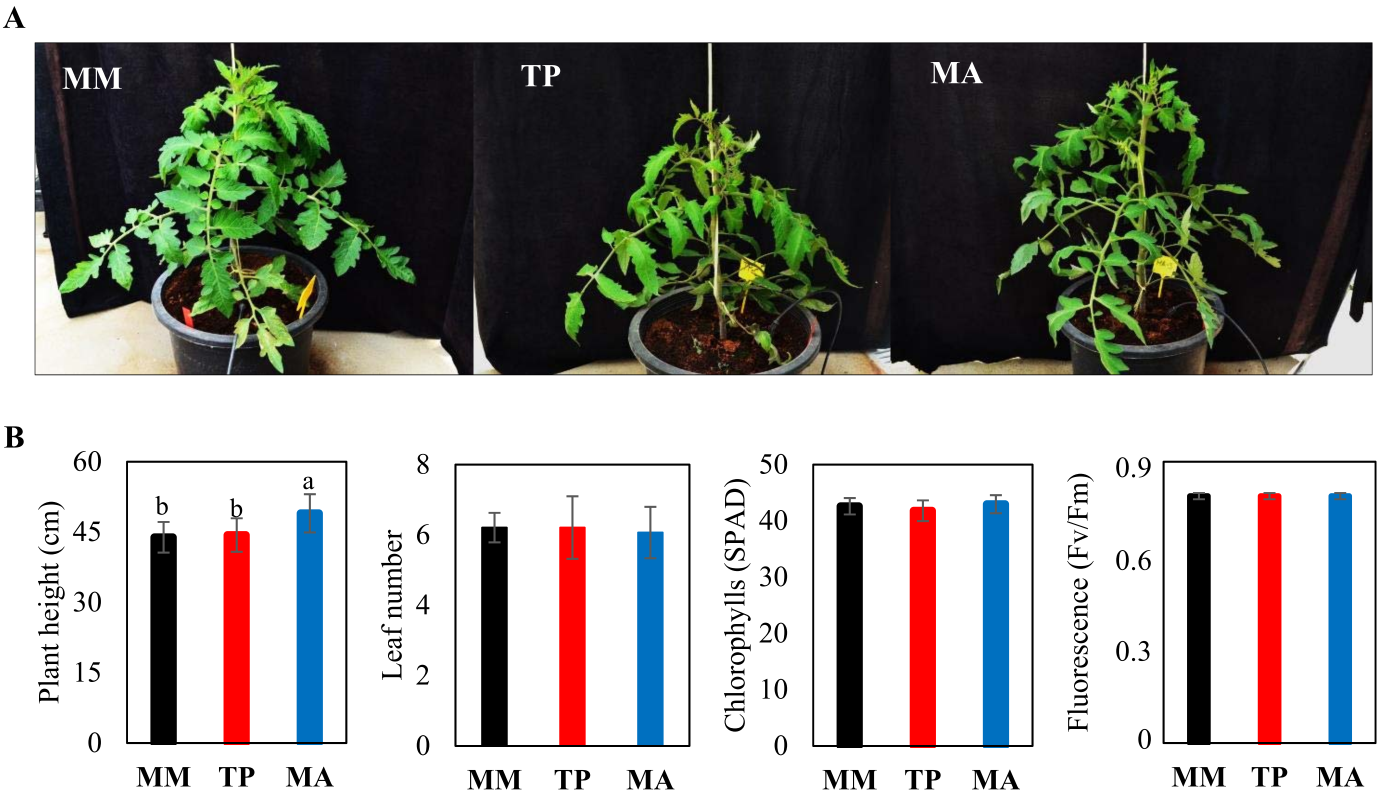

Supplementary Figure S1. Phenotypes of 'Moneymaker' (MM) and both traditional varieties plants, 'Tomate Pimiento' (TP) and 'Muchamiel Aperado' (MA) just before salt treatment (50 mM NaCl), after 45 days from sowing. **(A)** Representative images of plants and **(B)** traits of plant development (plant height and leaf number) and leaf chlorophyll content and fluorescence just before starting the salt treatment (50 mM NaCl). Values are expressed as means ± SE of fourteen plants per genotype. Different letters indicate statistically significant differences according to Tukey's test (p< 0.05).

Supplementary Table S1. Water percentage and cation contents (mg g^{-1} DW) in mature green and red ripe fruits of 'Moneymaker' and two traditional varieties, 'Tomate Pimiento' and 'Muchamiel Aperado', from plants grown in control and salt (50 mM NaCl).

	**Moneymaker**		**Tomate Pimiento**		**Muchamiel Aperado**	
	Control	Salt	Control	Salt	Control	Salt
			Mature green			
Water	94.7 ± 0.14^{a}	92.1 ± 0.09^{c}	93.2 ± 0.48^{b}	92.3 ± 0.09^{c}	94.1 ± 0.13^{a}	92.7 ± 0.14^{bc}
Na^{+}	0.39 ± 0.06^{c}	1.31 ± 0.32^{b}	0.60 ± 0.03^{c}	0.74 ± 0.03^{c}	1.39 ± 0.08^{b}	3.46 ± 0.19^{a}
K^{+}	33.0 ± 4.88^{a}	34.2 ± 8.45^{a}	34.4 ± 1.97^{a}	33.2 ± 1.22^{a}	40.9 ± 2.54^{a}	35.6 ± 2.09^{a}
Ca^{2+}	1.09 ± 0.17^{bc}	0.77 ± 0.05^{c}	1.33 ± 0.06^{ab}	1.07 ± 0.07^{bc}	1.17 ± 0.04^{bc}	1.57 ± 0.26^{a}
Mg^{2+}	0.86 ± 0.13^{b}	0.78 ± 0.19^{b}	1.35 ± 0.08^{a}	1.07 ± 0.05^{ab}	1.36 ± 0.09^{a}	1.26 ± 0.07^{a}
Ca^{2+}/Mg^{2+}	1.27 ± 0.01^{a}	0.99 ± 0.06^{b}	0.99 ± 0.01^{b}	1.00 ± 0.03^{b}	0.86 ± 0.04^{b}	1.24 ± 0.14^{a}
**Total cations**	35.4 ± 5.24^{a}	36.9 ± 9.14^{a}	37.7 ± 2.15^{a}	36.1 ± 1.36^{a}	44.9 ± 2.77^{a}	41.9 ± 2.59^{a}
			Red ripe			
Water	93.6 ± 0.04^{ab}	91.7 ± 0.46^{d}	93.0 ± 0.08^{bc}	91.1 ± 0.27^{d}	93.8 ± 0.05^{a}	92.7 ± 0.15^{c}
Na^{+}	0.44 ± 0.05^{cd}	1.03 ± 0.09^{b}	0.39 ± 0.01^{d}	0.84 ± 0.08^{bc}	1.08 ± 0.23^{b}	3.14 ± 0.26^{a}
K^{+}	35.7 ± 2.01^{a}	35.4 ± 2.91^{a}	35.4 ± 0.85^{a}	31.1 ± 3.10^{a}	38.2 ± 3.08^{a}	36.2 ± 2.57^{a}
Ca^{2+}	1.26 ± 0.14^{b}	1.72 ± 0.15^{a}	0.85 ± 0.04^{c}	1.01 ± 0.09^{bc}	1.17 ± 0.24^{bc}	1.12 ± 0.07^{bc}
Mg^{2+}	0.95 ± 0.05^{a}	1.01 ± 0.08^{a}	1.17 ± 0.03^{a}	1.11 ± 0.11^{a}	1.33 ± 0.29^{a}	1.15 ± 0.09^{a}
Ca^{2+}/Mg^{2+}	1.33 ± 0.08^{b}	1.70 ± 0.00^{a}	0.73 ± 0.01^{e}	0.91 ± 0.01^{cd}	0.88 ± 0.00^{d}	0.97 ± 0.01^{c}
**Total cations**	38.4 ± 2.24^{a}	39.1 ± 3.24^{a}	37.8 ± 0.94^{a}	34.0 ± 3.39^{a}	41.8 ± 3.85^{a}	41.6 ± 3.01^{a}

Values are expressed as means ± SE of three biological replicates of 10 fruits each. Different letters indicate statistically significant differences according to Tukey's test ($p < 0.05$).

Supplementary Table S2. Carotenoids and chlorophylls contents (µg g^{-1} DW) in mature green and red ripe fruits of 'Moneymaker' and two traditional varieties, 'Tomate Pimiento' and 'Muchamiel Aperado', from plants grown in control and salt (50 mM NaCl).

	**Moneymaker**		**Tomate Pimiento**		**Muchamiel Aperado**	
	Control	Salt	Control	Salt	Control	Salt
			Mature green			
Lycopene	ND	ND	ND	ND	ND	ND
β-Carotene	54.1±4.23^{a}	38.6±2.71^{b}	49.8±5.10^{a}	34.8±5.77^{b}	38.1±0.91^{b}	32.3±3.25^{b}
Lutein	20.2±2.91^{ab}	16.9±3.08^{abc}	22.8±2.09^{a}	15.7±1.96^{bc}	14.0±0.98^{c}	15.2±1.16^{bc}
Phytoene	20.4±1.07^{a}	17.1±0.97^{a}	19.4±2.15^{a}	18.7±1.54^{a}	18.3±2.36^{a}	17.0±1.05^{a}
Violaxanthin	21.7±1.91^{b}	11.2±1.70^{d}	28.8±2.80^{a}	17.3±1.18^{bc}	15.2±2.49^{cd}	11.9±1.94^{cd}
Neoxanthin	8.05±0.77^{a}	6.21±0.40^{b}	7.95±0.80^{a}	7.02±0.45^{ab}	7.29±0.85^{ab}	5.92±0.30^{b}
Chlorophyll a	377.7±12.8^{b}	279.2±11.5^{d}	319.1±2.90^{c}	429.2±5.18^{a}	234.4±6.89^{e}	252.4±5.40^{e}
Chlorophyll b	152.0±2.23^{a}	101.2±9.81^{cd}	119.8±10.8^{bc}	81.0±7.38^{e}	121.8±1.29^{b}	97.6±5.76^{de}
			Red ripe			
Lycopene	1564.6±126.5^{ab}	1442.2±109.4^{bc}	1790.7±67.5^{a}	1467.7±64.4^{b}	1233.0±73.2^{cd}	1084.6±6.61^{d}
β-Carotene	110.0±9.08^{a}	97.7±13.3^{ab}	93.7±11.6^{ab}	72.9±16.8^{bc}	55.1±5.65^{c}	54.6±4.40^{c}
Lutein	12.9±1.16^{a}	10.4±3.75^{ab}	8.53±0.61^{ab}	8.20±1.56^{ab}	6.76±1.01^{b}	6.42±0.55^{b}
Phytoene	138.1±7.67^{cd}	173.5±18.1^{bc}	217.5±27.2^{ab}	102.2±22.5^{d}	234.3±26.2^{a}	171.8±4.50^{bc}
Violaxanthin	ND	ND	ND	ND	ND	ND
Neoxanthin	ND	ND	ND	ND	ND	ND
Chlorophyll a	9.47±0.03^{bc}	3.51±0.10^{d}	15.3±1.47^{a}	7.70±1.01^{c}	10.6±2.08^{bc}	11.1±0.20^{b}
Chlorophyll b	15.2±0.05^{bc}	4.34±0.43^{d}	19.0±1.30^{a}	12.3±0.05^{c}	14.2±2.58^{c}	17.8±0.32^{ab}

Values are expressed as means ± SE of three biological replicates of 10 fruits each. Different letters indicate statistically significant differences according to Tukey's test ($p < 0.05$).
